# Response to the Letter to the Editor Entitled “The Weight of Disease, the Loss Within: Rethinking Frailty Assessment in ADPKD”

**DOI:** 10.1016/j.ekir.2026.106473

**Published:** 2026-03-10

**Authors:** Jui Wang, Szu-Ying Lee, Chia-Ter Chao

**Affiliations:** 1 Institute of Epidemiology and Preventive Medicine, College of Public Health, National Taiwan University, Taipei, Taiwan; 2 Health Data Research Center, National Taiwan University, Taipei, Taiwan; 3 Division of Nephrology, Department of Internal Medicine, National Taiwan University Hospital Yunlin Branch, Yunlin County, Taiwan; 4 Division of Nephrology, Department of Internal Medicine, National Taiwan University Hospital and National Taiwan University College of Medicine, Taipei, Taiwan; 5 Graduate Institute of Toxicology, National Taiwan University College of Medicine, Taipei, Taiwan; 6 Graduate Institute of Medical Education and Bioethics, National Taiwan University College of Medicine, Taipei, Taiwan

The Author Replies:

We thank Paolisi *et al.*^1^ for their thoughtful comments on our study examining frailty among patients with autosomal dominant polycystic kidney disease (ADPKD).^2^ We appreciate their recognition of our methodological approach and their emphasis on potential disease-specific heterogeneity. We have the following responses to their insightful comments.

First, we fully agree that ADPKD is genetically heterogeneous, with *PKD1* truncating variants associated with more severe phenotypes than *PKD2* pathogenic variants. Because genotypic data were unavailable in our database, as already acknowledged in the limitation section of our article,^2^ we primarily addressed ADPKD as encountered in routine clinical practice, during which genetic information is often unclear because of various reasons. Our findings therefore reflect population-level risk within a real-world cohort carefully matched for age, kidney function, comorbidities, medications, and baseline frailty status. Although it is plausible that high-risk genotypic subgroups may carry differential vulnerability, our results indicate that ADPKD was not independently associated with incident or worsening frailty during 4.8 years of follow-up, for those of middle age and with predominantly early-stage CKD (1 and 2). We agree that future genetically stratified studies would provide in-depth data for addressing this issue.

We also acknowledge the potential influence of organomegaly and volumetric burden on our results. Gastrointestinal compression and nutritional compromise represent biologically plausible pathways to sarcopenia development in kidney patients.^3^ However, in our matched cohort, baseline body mass index, laboratory indices, and FRAIL scores were comparable between groups. Importantly, worsening frailty was defined by longitudinal change across multiple FRAIL domains, not weight loss alone. Even if organ mass confounded the weight component, divergence in fatigue, resistance, ambulation, or illness burden would be expected if clinically meaningful sarcopenia were prevalent at a population level. Nonetheless, such divergence was not observed in this study.

Finally, we believe that the observed increase in mortality without increased frailty risk may reflect the effects of other ADPKD-specific complications, such as vascular or cyst events, rather than unrecognized global vulnerability. This distinction underscores that adverse outcomes in ADPKD may be mediated through disease-specific mechanisms not fully captured by traditional frailty constructs that put emphasis on physical domains. In addition, it is plausible that other multidimensional frailty assessment tools can facilitate the identification of mediators for adverse outcomes in patients with ADPKD.^4^

In conclusion, we concur that future investigations incorporating genetic diagnostics, volumetric imaging, body composition assessment, and multidimensional frailty tools will further clarify functional trajectories in ADPKD. We are grateful for the opportunity to advance this discussion.

## Disclosure

All the authors declared no competing interests.
